# Dynamic community detection reveals transient reorganization of functional brain networks across a female menstrual cycle

**DOI:** 10.1162/netn_a_00169

**Published:** 2021-02-01

**Authors:** Joshua M. Mueller, Laura Pritschet, Tyler Santander, Caitlin M. Taylor, Scott T. Grafton, Emily Goard Jacobs, Jean M. Carlson

**Affiliations:** Interdepartmental Graduate Program in Dynamical Neuroscience, University of California, Santa Barbara, Santa Barbara, CA, USA; Department of Physics, University of California, Santa Barbara, Santa Barbara, CA, USA; Department of Psychological and Brain Sciences, University of California, Santa Barbara, Santa Barbara, CA, USA; Department of Psychological and Brain Sciences, University of California, Santa Barbara, Santa Barbara, CA, USA; Department of Psychological and Brain Sciences, University of California, Santa Barbara, Santa Barbara, CA, USA; Interdepartmental Graduate Program in Dynamical Neuroscience, University of California, Santa Barbara, Santa Barbara, CA, USA; Department of Psychological and Brain Sciences, University of California, Santa Barbara, Santa Barbara, CA, USA; Interdepartmental Graduate Program in Dynamical Neuroscience, University of California, Santa Barbara, Santa Barbara, CA, USA; Department of Psychological and Brain Sciences, University of California, Santa Barbara, Santa Barbara, CA, USA; Neuroscience Research Institute, University of California, Santa Barbara, Santa Barbara, CA, USA; Interdepartmental Graduate Program in Dynamical Neuroscience, University of California, Santa Barbara, Santa Barbara, CA, USA; Department of Physics, University of California, Santa Barbara, Santa Barbara, CA, USA

**Keywords:** Sex hormones, Dynamic community detection, Dense sampling, Network flexibility

## Abstract

Sex steroid hormones have been shown to alter regional brain activity, but the extent to which they modulate connectivity within and between large-scale functional brain networks over time has yet to be characterized. Here, we applied dynamic community detection techniques to data from a highly sampled female with 30 consecutive days of brain imaging and venipuncture measurements to characterize changes in resting-state community structure across the menstrual cycle. Four stable functional communities were identified, consisting of nodes from visual, default mode, frontal control, and somatomotor networks. Limbic, subcortical, and attention networks exhibited higher than expected levels of nodal flexibility, a hallmark of between-network integration and transient functional reorganization. The most striking reorganization occurred in a default mode subnetwork localized to regions of the prefrontal cortex, coincident with peaks in serum levels of estradiol, luteinizing hormone, and follicle stimulating hormone. Nodes from these regions exhibited strong intranetwork increases in functional connectivity, leading to a split in the stable default mode core community and the transient formation of a new functional community. Probing the spatiotemporal basis of human brain–hormone interactions with dynamic community detection suggests that hormonal changes during the menstrual cycle result in temporary, localized patterns of brain network reorganization.

## Author Summary

Sex steroid hormones influence the central nervous system across multiple spatiotemporal scales. Estrogen and progesterone concentrations rise and fall throughout the menstrual cycle, but it remains poorly understood whether day-to-day fluctuations in hormones shape human brain dynamics. Here, we assessed the structure and stability of resting-state brain network connectivity in concordance with serum hormone levels from a female who underwent fMRI and venipuncture for 30 consecutive days. Our results reveal that while network structure is largely stable over the course of a menstrual cycle, temporary reorganization of several large-scale functional brain networks occurs during the ovulatory window. In particular, a default mode subnetwork exhibits increased connectivity with itself and with nodes belonging to the temporoparietal and limbic networks, providing novel perspective into brain-hormone interactions.

## INTRODUCTION

The application of network science techniques to the study of the human brain has revealed a set of large-scale functional brain networks that meaningfully reorganize both intrinsically and in response to external task demands (Bassett & Sporns, 2017). One technique, dynamic community detection (DCD), has emerged as a powerful tool for conceptualizing and quantifying changes in mesoscale brain network connectivity patterns by identifying sets of nodes (communities) with strong intracommunity connections (Newman, 2006) to enable identification of communities that persist or change over time. DCD complements other statistical approaches used in fMRI data analysis by identifying when functionally coupled brain regions undergo sufficiently large changes in connectivity to warrant reassignment to separate functional communities. Additionally, this method provides an interpretable summary of whether strongly connected sets of brain regions undergo transient, but significant, changes that could be missed when time-averaging data within and between sessions.

This method is particularly suited for examining relationships between brain dynamics and physiological variables that vary over relatively short timescales, such as sex hormone fluctuations over the human menstrual cycle. A typical cycle, occurring every 25–30 days, is characterized by significant rises in estradiol (∼8-fold) and progesterone (∼80-fold), both of which are powerful neuromodulators that have a widespread influence on the central nervous system (Galea, Frick, Hampson, Sohrabji, & Choleris, 2017). Converging evidence from animal studies has established sex hormones’ influence on regions supporting higher order cognition, including the prefrontal cortex (PFC) and hippocampus (Frick, 2015; Wang, Hara, Janssen, Rapp, & Morrison, 2010). Within these regions, for example, estradiol enhances spinogenesis and synaptic plasticity while progesterone largely abolishes this effect (Hara, Waters, McEwen, & Morrison, 2015; Woolley & McEwen, 1993). Importantly, sex hormones are expressed broadly throughout the cerebellum and cerebrum, suggesting that whole-brain effects might be observed beyond the regions targeted in these preclinical studies.

Human neuroimaging studies have demonstrated that sex hormones influence brain activity across broad regions of cortex (Berman et al., 1997; Jacobs & D’Esposito, 2011). Further, a handful of studies have demonstrated that menstrual cycle stage shapes resting-state functional connectivity (rs-fc; Arélin et al., 2015; Lisofsky et al., 2015; Petersen, Kilpatrick, Goharzad, & Cahill, 2014; Weis, Hodgetts, & Hausmann, 2019). However, these studies typically involve group-based cross-sectional studies or sparse-sampling (two–four time points) designs that are unable to capture transient day-to-day relationships between sex hormones and functional brain dynamics, and this relatively low temporal resolution has led to inconsistencies in the literature (Hjelmervik, Hausmann, Osnes, Westerhausen, & Specht, 2014). Therefore, new approaches are needed that can address these spatial and temporal limitations, as doing so will provide novel perspectives on human brain-hormone interactions.

**Figure 1.**
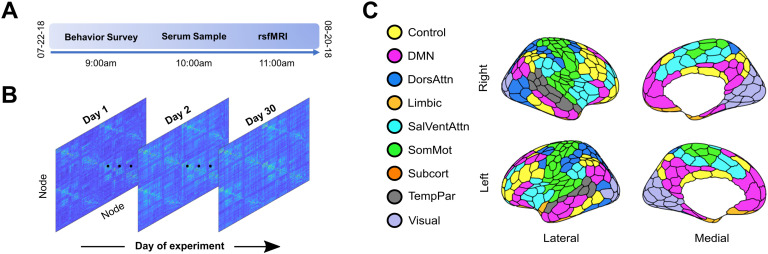
28andMe dataset. (A) Subject LP (naturally cycling female, age 23) participated in a month-long “dense-sampling” experimental protocol to provide a multimodal, longitudinal dataset referred to as 28andMe (Pritschet et al., 2020). For 30 consecutive days, the subject completed assessments of diet, mood, and sleep, provided blood samples to examine serum hormone concentrations, and underwent a 10-minute resting-state fMRI scan. (B) For each resting-state scan, functional connectivity matrices were constructed by calculating the pairwise mean magnitude-squared coherence between each region. The result is a 415 × 415 × 30 data structure, in which each entry indicates the coherence between two nodes on a given day. (C) The brain was parcellated into 415 regions that were assigned to one of nine networks based on previously identified anatomical and functional associations (Schaefer et al., 2018). Colors indicate regional network membership. In a follow-up experiment, the participant repeated the procedures while on a hormonal regimen (0.02 mg ethinyl-estradiol, 0.1 mg levonorgestrel, Aubra, Afaxys Pharmaceuticals), which she began 10 months prior to the start of data collection (Pritschet et al., 2020; Taylor et al., 2020).

Recently, Pritschet et al. (2020) applied a “dense-sampling” approach (Laumann et al., 2015; Poldrack, Laumann, & Koyejo, 2015) to a naturally cycling female who underwent 30 consecutive days of brain imaging and venipuncture to capture rs-fc variability over a complete menstrual cycle (Figure 1). The authors found that day-to-day fluctuations in estradiol were associated with widespread increases in rs-fc across the whole brain, with progesterone showing an opposite, negative relationship. Using time series modeling and graph theoretical analysis, they also found that estradiol drives variation in topological network states, specifically within-network connectivity of default mode and dorsal attention networks. These findings have important implications for the field of network neuroscience where dense-sampling, deep-phenotyping approaches have emerged to aid in understanding sources of intra/inter-individual variability in functional brain networks over days, weeks, months, and years (Chen et al., 2015; Gratton et al., 2018; Poldrack et al., 2015).

Pritschet and colleagues’ approach identified node-averaged trends in rs-fc changes within canonical functional networks across the cycle, but questions remain regarding whether and where functional reorganization takes place between large-scale networks. As changes in edge weight can result in the formation of functional “communities” not captured by traditional rs-fc methods, complementary approaches are needed to characterize trends in brain connectivity at intermediate spatial and temporal scales. Examining mesoscale networks has further revealed fundamental principles of functional brain networks, such as the modular, integrated architecture underpinning flexible task performance (Bertolero, Yeo, & D’Esposito, 2015; Khambhati, Sizemore, Betzel, & Bassett, 2018). Additionally, a better understanding of mesoscale connectivity may provide an avenue for improving personalized medicine by increasing the efficacy of targeted therapeutic interventions (Gu et al., 2015).

Here, we applied DCD to examine whole-brain dynamics in relation to sex hormone fluctuations across a menstrual cycle. Our results reveal that a stable set of “core” communities persist over the course of a menstrual cycle, primarily consisting of nodes belonging to distinct a priori defined functional–anatomical networks, namely visual, somatomotor, attention, default mode, and control networks. Though these core communities were largely stable, nodes belonging to limbic, subcortical, attention, and control networks changed community affiliation (referred to as flexibility) at higher rates than expected compared with a null hypothesis.

DCD also identified a transient split of the default mode network (DMN) core into two smaller subcommunities concurrent with peaks in estradiol, luteinizing hormone (LH), and follicle stimulating hormone (FSH) levels defining the ovulatory window. This community split was driven by strong increases of within-network integration between prefrontal nodes of the DMN, which subsided immediately after the ovulatory window. The default mode, temporoparietal, limbic, and subcortical networks also exhibited significantly increased flexibility during ovulation, suggesting a role for estradiol, LH, and FSH in regulating localized, temporary changes in regional connectivity patterns. Importantly, this reorganization was not present in a follow-up study in which the same participant was placed on an oral hormonal regimen that prevented ovulation. Taken together, while a large degree of functional brain network stability was observed across the menstrual cycle, peaks in sex hormones resulted in temporary brain network reorganization, suggesting that sex hormones may have the ability to rapidly modulate rs-fc on shorter timescales than previously documented.

## RESULTS

A single female underwent brain imaging and venipuncture for 30 consecutive days. For each session, the brain was parcellated into 400 cortical regions from the Schaefer atlas and 15 subcortical regions from the Harvard–Oxford atlas (Figure 1C) and 415 × 415 functional association matrices were constructed via magnitude-squared coherence (Schaefer et al., 2018). Dynamic community detection was applied to these data, revealing a stable set of communities that persist over the course of a menstrual cycle. However, significant transient changes in community structure occurred within the default mode network during the ovulatory window concomitant with peaks in estradiol, luteinizing hormone, and follicle stimulating hormone.

### Stable Functional Cores Persisted Over the Course of One Menstrual Cycle

**Figure 2.**
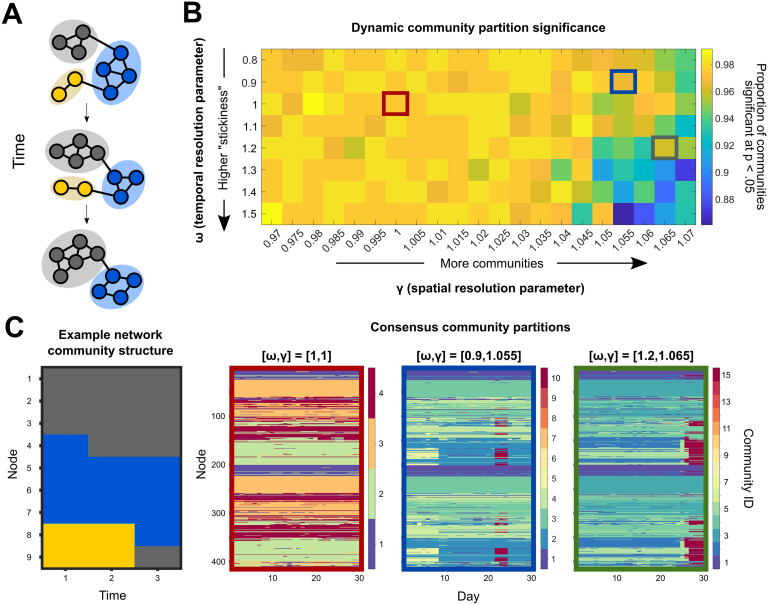
Dynamic community detection identified changing modular structure over time at multiple scales. (A) A toy network example illustrates the dynamic community detection algorithm. For each time point, every node is assigned to a community so as to maximize the strength of intracommunity connections relative to intercommunity links while also taking community assignments over time into account (Eq. 1). In this case, three communities are identified and denoted by color. (B) To assess temporal structure in the 28andMe resting-state fMRI data, community assignments were calculated for a range of parameter values. In this procedure, two parameters, *ω* and *γ*, specify the temporal and spatial scales of analysis, respectively. After performing 150 runs of the community detection algorithm for each parameter combination, the statistical significance of each community partition relative to a random null model was calculated. The color for each entry in the heat map indicates the proportion of communities at that parameter combination that are significant at the *p* < 0.05 level. (C) Consensus partition structure varied according to the choice of resolution parameters. The example network community structure (left) changes at each time point, with node community assignment given by color on the y-axis and time indicated on the x-axis. For three different parameter combinations (outlined in red, blue, and green, respectively), the consensus partitions varied in the total number of communities identified, ranging from 4 to 15, with more communities identified when the temporal resolution was low and the spatial resolution was high.

The degree to which functional brain network connectivity changes over the course of a human menstrual cycle has yet to be fully characterized. Here, dynamic community detection (also referred to as multislice or multilayer modularity maximization (Bassett et al., 2013)) consistently identified four functional communities that were largely stable in a naturally cycling female over 30 consecutive days. In this context, “community” refers to a set of nodes whose intraset connections are significantly stronger than would be expected when compared with an appropriate null model. A representative example of this consensus temporal community structure (the community designation that best matches the output of 150 runs of the nondeterministic community detection algorithm) is shown in Figure 2C. This structure was conserved over a range of community detection parameter values that, roughly speaking, must be defined to set the “spatial“ and “temporal” resolutions of community identification (see the Methods section for a detailed description). Across all temporal resolutions considered here, consensus community partitions with a spatial resolution parameter 0.97 ≤ *γ* ≤ 1.015 possessed exactly four communities.

**Figure 3.**
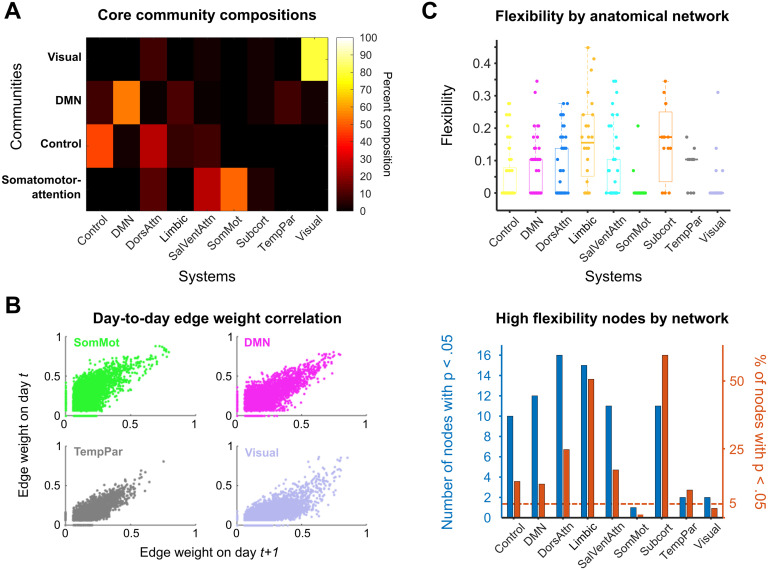
Dynamic community detection uncovered stable cores across a complete menstrual cycle. (A) Four core communities (*y*-axis) were consistently identified in the 28andMe dataset across spatial and temporal resolution parameter values. For these parameter combinations, the compositions of the visual, default mode, control, and somatomotor-attention network cores are shown as a heat map, with color corresponding to the percentage of nodes in a community belonging to a functional–anatomical network. (B) The four networks that constituted the hubs of the core communities possessed stable pairwise connectivity between nodes across days. Scatterplots show the day-to-day correspondence between edge weights for all of the nodes of the somatomotor, default mode, temporoparietal, and visual networks on days *t* and *t* + 1. These network edges had Pearson correlation coefficients of 0.379, 0.573, 0.590, and 0.538, respectively. (C) The subcortical, limbic, and dorsal attention networks exhibited the highest median node flexibility. Top: Normalized flexibility values for each node over the entire cycle are plotted as points, with color indicating network affiliation. Thick horizontal lines on box plots indicate median values. A flexibility value of 1 indicates that a node changes community assignment at each possible time point, whereas a value of 0 indicates that the node never changes community assignment. Bottom: A 95% cutoff value is calculated using the flexibility values for each node over all 150 community detection runs. For each functional–anatomical network, the blue bar indicates the number of nodes belonging to that network which have flexibility values above the cutoff threshold. The red bars indicate the proportion of nodes in each network that surpass the cutoff value (i.e., the value for each blue bar is normalized by the number of nodes in the network). Once again, limbic, subcortical, dorsal attention, and control networks contained the highest proportion of highly flexible nodes.

For the standard parameter choice (temporal and spatial resolution parameters both set to 1), the four identified communities had distinct compositional characteristics. These communities were largely bilaterally symmetric, with analogous brain regions in each hemisphere assigned to the same community 71% of the time. The four communities correspond roughly to a visual core, a default mode core, a control core, and a somatomotor-attention core. The compositions of these four communities are shown in Figure 3A. The composition value was calculated by summing the total number of instances in which a node belonging to an a priori functional-anatomical network (Schaefer et al., 2018) also belonged to the community identified in the consensus community partition.

The core communities identified here were named based on the highest representation of nodes belonging to a priori functional networks. The visual core was 80% composed of visual network nodes and had a median size of 52 nodes per day. The default mode core consisted of 56% DMN nodes and approximately 10% of each control, limbic, and temporoparietal network nodes and contained a median of 133.5 nodes per day. The control core consisted of 48% control and 28% dorsal attention network nodes and contained a median of 133 nodes per day. Finally, the somatomotor-attention core was composed of 53% somatomotor, 27% salience-ventral attention, and 13% dorsal attention network nodes and had a median size of 97 nodes per day. Importantly, for all parameter combinations in which four communities were detected, the composition of these communities was consistent (Supporting Information). These community partitions were also stable across the entire menstrual cycle. Specifically, 315 of the 415 nodes (75.9%) did not change community affiliation across the 30-day experiment.

Taken together, these results suggest the presence of a stable solution to the dynamic community detection algorithm and a reliable coarse-grained community architecture present in the data. In several functional–anatomical networks, there was little to no modification of network architecture over time; for instance, greater than 85% of nodes in each of the somatomotor, default mode, temporoparietal, and visual networks did not change community affiliation over the entire menstrual cycle. The strong day-to-day correlations between edge weights in these networks (Figure 3B) reinforce the existence of these stable cores.

### Functional–Anatomical Networks Exhibited Distinct Patterns of Flexibility

Though network community structure was stable over a complete menstrual cycle when classifying nodes into four communities, specific nodes did change community affiliation at levels above chance when modifying the sensitivity of the community detection algorithm. Specifically, when *γ*, the spatial resolution parameter, was increased, the dynamic community detection algorithm subdivided the four core communities into smaller communities, providing a finer grained classification of subnetwork structure. At an intermediate parameter combination (*ω* = 0.9, *γ* = 1.055), 10 communities significant at the *p* < 0.05 level were identified over the course of the experiment, as visualized in Figure 2C (blue outlines). The subsequent analysis uses community partitions at this parameter combination, but the results were consistent across a range of neighboring parameter values (Supporting Information).

This “higher resolution” partition revealed trends in functional organization over time that were not observable with coarser partitions. First, inspecting the median flexibility value, or the proportion of times a node changed community affiliation out of the total possible number of changes, demonstrates that functional–anatomical networks possessed distinct flexibility distributions (Figure 3C, top). The limbic, subcortical, dorsal attention, and control networks were significantly overrepresented in terms of highly flexible nodes relative to a random null hypothesis (Figure 3C, bottom).

**Figure 4.**
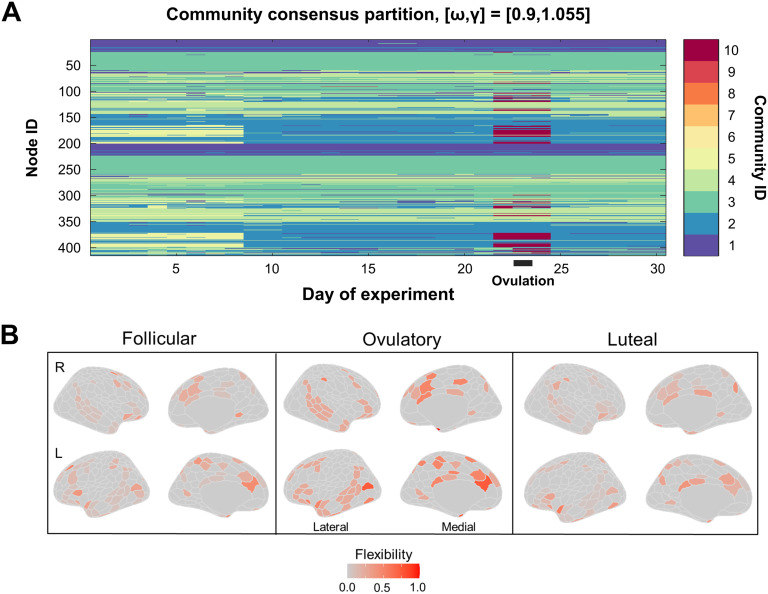
Fine-grain community partitioning revealed a bifurcation in the default mode core during ovulation. (A) When the spatial resolution parameter (which alters the size of communities identified by dynamic community detection) was increased from the standard value, the four core communities identified previously were subdivided into smaller subcommunities (reproduced from Figure 2C). Here, a split in the default mode core community (light blue) appeared at Day 22 (red), concomitant with ovulation and a spike in sex hormones. This community (red) rejoined the default mode core on day 26. For illustrative purposes, only the consensus partition for one parameter value is shown, but this trend was consistent across nearby parameter combinations (Supporting Information). (B) Shown are flexibility values for each node by menstrual cycle phase. Color in each region indicates flexibility value, with hotter colors indicating higher values. The following days of the experiment corresponded to the phases of the menstrual cycle: follicular, Days 11–22; ovulatory, Days 23–25; luteal, Days 1–10 and 26–30. Flexibility values are noticeably higher in many regions from the temporoparietal, limbic, subcortical, and default mode networks during the ovulatory phase compared with the follicular and luteal phases. Mowinckel, A.M. and Vidal-Piñeiro, D. (2019) Visualisation of Brain Statistics with R-packages ggseg and ggseg3d. arXiv:stat.OT/1912.08200.

The largest fine-scale community reorganization occurred on experiment Day 22 and persisted until Day 24 (Figure 4A). Across these days, 65 nodes belonging to the default mode core community split from the default mode core community to transiently form a small, strongly connected community. This was one of only two large-scale reorganization events detected during the experiment; the other occurred on Day 9, when 59 nodes (yellow in Figure 4A) changed community affiliation. All 59 nodes involved in this event also changed community affiliation on Day 22.

**Figure 5.**
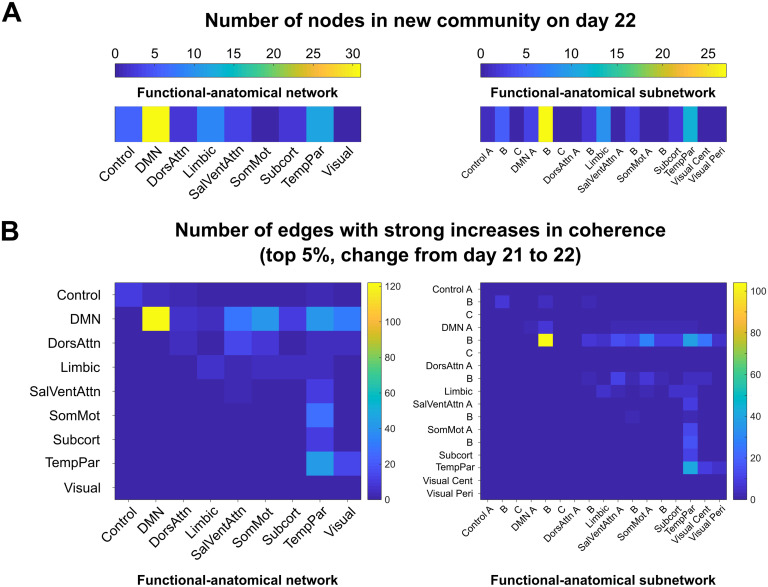
Nodes in a default mode subnetwork drove community bifurcation via strong increases in coherence. (A) The newly formed functional community on Days 22–24 contained 65 nodes that belonged to the community on all three days. The functional–anatomical network and subnetwork affiliations of these nodes are shown on the left and right, respectively. The new community contained 31 DMN nodes, 12 temporoparietal nodes, and 9 limbic nodes. (B) The edges that exhibited large weight changes from Day 21 to Day 22 (top 5% of changes, left) were predominantly within-network connections between DMN network nodes (104/466). Examining subnetwork structure reveals that all of the strongly enhanced connections between nodes in the DMN belonged to subnetwork B, indicating that this subnetwork, which consists of regions in prefrontal cortex, drove the default mode core community bifurcation at ovulation.

Interestingly, 31 (48%) of the nodes in the community that emerged on Day 22 belonged to the DMN, 12 nodes (19%) belonged to the temporoparietal network, and 9 (14%) were limbic regions (as defined by functional–anatomical atlases (Jenkinson, Beckmann, Behrens, Woolrich, & Smith, 2012; Schaefer et al., 2018; Figure 5A). The functional–anatomical network memberships of the node–node pairs exhibiting the strongest increases in coherence (top 5%) indicated that enhanced connectivity between DMN nodes drove this community split, as opposed to DMN nodes being “converted” to a new community via increased connectivity to non-DMN regions (Supporting Information). More specifically, nodes within prefrontal regions belonging to DMN subnetwork B drove this reorganization event, as 104 of the 466 (22%) strongest increases in coherence occurred between nodes in this subnetwork, despite DMN subnetwork B containing only 32 nodes (8% of the total nodes).

### Network Reorganization Timing Coincided With Peaks in Hormone Levels During Ovulation

**Figure 6.**
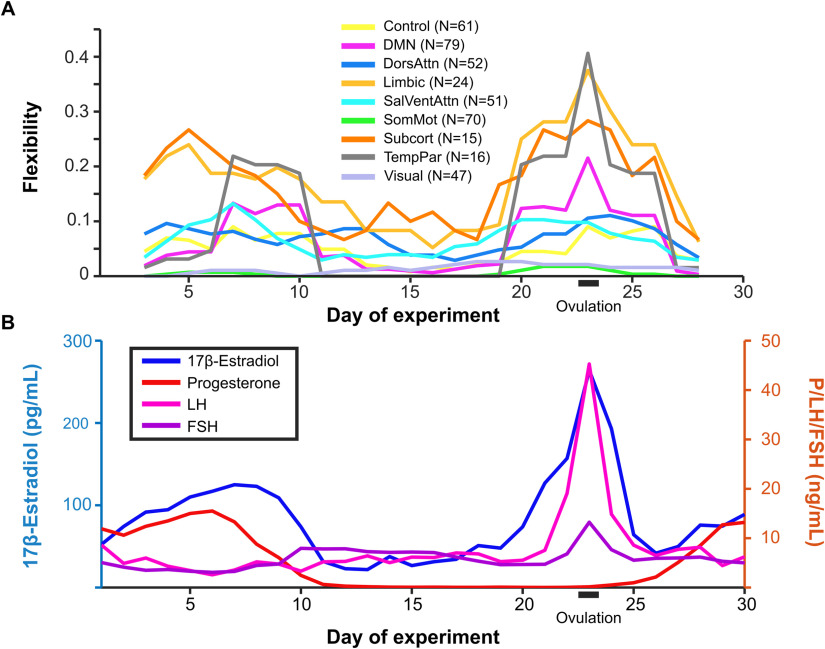
Community reorganization was temporally localized to ovulation. Changes in community assignment (A) were coordinated and closely tracked the timing of spikes in estradiol concentrations (B). Default mode, limbic, subcortical, and temporoparietal networks exhibited peaks in flexibility on Day 23, indicating brain-wide functional reorganization during the ovulatory window. These same networks also exhibited elevated flexibility between Days 5 and 10 during the secondary estradiol peak. The pattern of flexibility shown here corresponds to the network reorganization observed for dynamic community detection performed with the parameter combination *ω* = 0.9, *γ* = 1.055 (blue outline in Figure 2). Here, flexibility is calculated over a five-day sliding window.

Global flexibility was higher (Wilcoxon rank-sum test, *p* < 0.05) during ovulation (Days 23–25) than during early follicular or luteal phases. Specifically, global mean flexibility during the ovulatory window was 0.10, whereas flexibility during follicular and luteal phases was 0.05 and 0.04, respectively. Flexibility of individual brain regions during these phases are shown in Figure 4B. Note that while several nodes exhibit high flexibility across all three phases, global flexibility and network-specific mean flexibility are still relatively low (as seen in Figures 3C and Figure 6A) because the majority of nodes rarely change community affiliation.

Mean flexibility of each network over a five-day sliding window is depicted in Figure 6A. The DMN, temporoparietal, subcortical, and limbic networks exhibited peaks in flexibility at Day 23 of the experiment, coincident with the peaks in estradiol, LH, and FSH which are a hallmark signals of the ovulatory window (Figure 6B). To determine whether the bifurcation of the default mode core community was significantly associated with sex hormones, we compared functional–anatomical network flexibility values with serum hormone levels.

To assess the temporal relationship between network flexibility values and sex hormones, correlations between each time series were calculated. The default mode, limbic, salience/ventral attention, somatomotor, subcortical, and temporoparietal networks had significant Spearman rank correlations greater than 0.6 (where maximum value of 1 indicates perfect rank correlation and 0 indicates no correlation) with estradiol (Bonferroni-corrected at *p* < 0.05). No other significant positive network flexibility-hormone correlations were identified (Supporting Information).

Next, to determine whether these reorganization events are uniquely related to the intrinsic hormonal dynamics that occur across a menstrual cycle, we conducted an identical analysis from a follow-up dataset in which the same individual repeated the daily protocol (30 consecutive days of sampling) one year later. During this follow-up study, the participant was placed on a hormonal regimen that disrupted endogenous sex hormone production and prevented ovulation from occurring (see Pritschet et al., 2020; Taylor et al., 2020). Under this regimen, DCD identified the same four stable cores found in the original experiment, but no large-scale reorganization was observed (Supporting Information).

## DISCUSSION

In this study, we applied DCD to data from a densely sampled female who underwent 30 consecutive days of brain imaging and venipuncture to investigate the extent of intrinsic spatiotemporal functional reorganization over a menstrual cycle. We identified four stable community cores across the cycle, represented here as visual, somatomotor, default mode, and control network cores; the strongest exception to this stability occurred simultaneously with peaks in estradiol, LH, and FSH. During this event, we observed a transient reorganization of the DMN core into a newly formed community, as well as increases in nodal flexibility among prefrontal, limbic, and subcortical nodes. A nearly identical reorganization event occurred during the secondary peak in estradiol. Together, our results suggest that the interplay between the nervous and endocrine systems over a menstrual cycle results in temporary, localized patterns of brain network reorganization. These results highlight DCD as a new avenue for investigating the intricate relationship between sex hormones and human brain dynamics.

### Dynamic Community Detection Characterizes Network-Specific Functional Stability Across a Menstrual Cycle

Dense-sampling, deep-phenotyping studies offer new ways to investigate intra/interindividual variability in functional brain networks by identifying features of rs-fc that are stable traits within an individual or change in conjunction with biological factors and state-dependent variables (Gratton et al., 2018; Poldrack et al., 2015). Recent dense-sampling studies have shown that frontoparietal regions/networks exhibit high degrees of intra-individual rs-fc stability while also being characteristically unique across individuals, suggesting that these higher order regions may be especially critical for uncovering individual differences in brain function and improving personalized medicine (Gratton et al., 2018; Horien, Shen, Scheinost, & Constable, 2019). Our findings provide new insight towards the ongoing explorations into stability within functional brain networks. With the exception of limbic and subcortical networks, network nodes were highly stable, changing affiliations fewer than 10% of the time on average (Figure 3C). Therefore, our results align with previous research suggesting a high degree of network stability in resting-state networks in individuals over time (Gratton et al., 2018; Hjelmervik et al., 2014; Horien et al., 2019; Poldrack et al., 2015).

In contrast to this observed overall stability, several highly flexible nodes were identified. Control subnetwork C, encompassing posterior cingulate cortex/precuneus regions, was the most flexible functional subnetwork identified, with 10 of the 12 nodes exhibiting significantly higher than expected flexibility (Supporting Information). Limbic and subcortical networks displayed intermediate levels of flexibility. Regions from these systems receive input from and project to many cortical areas and are implicated in functions such as sensorimotor integration via the cortico-basal ganglia-thalamo-cortical loop (Bell & Shine, 2016); therefore, the high degree of flexibility observed here may reflect the tendency of these systems to serve as relays between functionally segregated communities.

Particular changes in rs-fc were significantly related to sharp rises in sex hormones seen during ovulation. Here, we observed a spatially specific transient reorganization of the DMN, during which nodes from the temporoparietal, limbic, subcortical, and default mode networks split from the default mode core to form a short-lived community for (three days) before rejoining the original core community. Notably, a nearly identical event occurred on Day 9 of the experiment, when 59 of the 65 nodes that changed community affiliation during ovulation merged with the default mode core. This event occurred concurrent with a secondary peak in estradiol and involved networks (DMN, temporoparietal, limbic, subcortical) whose flexibility values were significantly associated with estradiol levels, further implicating hormone-specific modulation of functional connectivity between these networks.

Using time-lagged analyses, Pritschet and colleagues reported that within-network connectivity of the DMN was regulated by previous states of estradiol (Pritschet et al., 2020). Here, we expand on this finding and identify a subnetwork of the DMN that is likely driving this reorganization. Regions constituting this new community are located in PFC, an area exquisitely sensitive to sex steroid hormones (Shanmugan & Epperson, 2014) where, for instance, nearly 50% of pyramidal neurons in the dorsolateral PFC (dlPFC) express ER-alpha (Wang et al., 2010). Importantly, this coordinated reorganization was not observed in a follow-up experiment in which typical hormone production patterns were disrupted (Supporting Information). Together, this presents the possibility that endocrine signaling may, in part, regulate intrinsic brain dynamics within the frontal cortex.

### Neurobiological Interpretations of Sex Hormones on PFC Function

Cross-species investigations have established estrogen’s ability to shape the PFC (Galvin & Ninan, 2014; Hara et al., 2016; Jacobs & D’Esposito, 2011; Jacobs et al., 2017; Shanmugan & Epperson, 2014). In rodents, estradiol increases fast-spiking interneuron excitability in deep cortical layers (Clemens et al., 2019); in nonhuman primates, estradiol treatment increases dendritic spine density in dlPFC neurons (Hao et al., 2006) and this potentiation is observed only if the treatment is administered in the typical cyclical pattern observed across a menstrual cycle. Human brain imaging studies have also implicated estradiol in enhancing the efficiency of PFC-based circuits. In cycling women performing a working memory task, PFC activity is exaggerated under low estradiol conditions and reduced under high estradiol conditions (Jacobs & D’Esposito, 2011). Similarly, when estradiol declines across the menopausal transition, working memory–related PFC activity becomes more exaggerated despite no differences in task performance (Jacobs et al., 2017). Examining rs-fc across the cycle, Petersen and colleagues found that women in the late follicular stage (encompassing the ovulatory window) showed increased coherence within DMN and executive control networks compared with those in luteal stages (Petersen et al., 2014). Our findings extend this body of work by demonstrating that PFC nodal flexibility tracks significantly with sharp shifts in estradiol, which may support the brain’s ability to reorganize at the mesoscale level.

While future studies are needed to establish a mechanistic link between endocrine signaling and large-scale network reorganization, we present two possible neurobiological interpretations. One mechanism of action may be through estradiol’s interaction with the dopaminergic system. The PFC is innervated by midbrain dopaminergic neurons that enhance the signal-to-noise ratio of PFC pyramidal neurons and drive cortical efficiency (Williams & Goldman-Rakic, 1995). In turn, estradiol enhances dopamine release and modifies the basal firing rate of dopaminergic neurons, potentially having the ability to alter mesoscale network integration. Second, although coherence is robust to changes in the hemodynamic response (Sun, Miller, & D’Esposito, 2004), sex hormones influence cerebrovascular function (Krause, Duckles, & Pelligrino, 2006; Krejza, Rudzinski, Arkuszewski, Onuoha, & Melhem, 2013). Therefore, the observed changes in rs-fc across the cycle could be due to changes in perfusion rather than alterations in neural activity.

Important differences in network stability emerged between naturally cycling and oral hormonal contraceptive conditions. Under naturally cycling conditions, the largest reorganization event occurred during the ovulatory window. Although estradiol levels were comparable in Study 2 (Supporting Information), estradiol was decoupled from LH and FSH, progesterone was reduced by 97%, and ovulation did not occur. Therefore, hormone-related changes in DMN subnetwork reorganization might only be present when shifts in endogenous hormones occur in a coordinated fashion. Future studies comparing endocrine states of women over several cycles will help establish the robustness of these differences.

### Implications for Cognition and Disease

Several studies have begun utilizing DCD to relate “task-free“ and “task-based” functional network reorganization to cognitive performance. High levels of nodal flexibility have been associated with enhanced performance on working memory tasks (Braun et al., 2015), improved learning of a motor task (Bassett et al., 2011), and visual cue learning (Gerraty et al., 2018). Further, sensory regions typically participate in a small number of functional networks during various tasks, whereas “hub” regions in frontal cortex, including precuneus and posterior cingulate gyrus, participate in multiple functional networks (van den Heuvel & Sporns, 2013), indicating that network-specific temporal reconfiguration of functional connectivity has implications for a wide variety of cognitive functions (Mattar, Cole, Thompson-Schill, & Bassett, 2015).

Highly flexible nodes were identified in precuneus and posterior cingulate gyrus, with changes in community affiliation occurring simultaneously with sharp peaks in estradiol levels, raising the possibility that hormonal fluctuations could be associated with and facilitate task-based network reorganization. For instance, if high levels of estradiol increase nodal flexibility among hub regions in the PFC, one might predict that performance on PFC-dependent tasks will improve. Further, pregnancy—a period of profound neuroendocrine change—leads to long-lasting gray matter reductions within DMN regions (Hoekzema et al., 2017). The capacity for the brain to fluctuate between integrated and segregated states at rest allows for rapid and efficient transitions to various task states (Kabbara, Falou, Khalil, Wendling, & Hassan, 2017; Shine et al., 2016; Zalesky, Fornito, Cocchi, Gollo, & Breakspear, 2014). Therefore, future work examining whether task-based functional brain networks undergo transient changes in flexibility and community structure, both across the menstrual cycle and during other hormonal transition periods, will be imperative.

Examining how large-scale brain networks are disrupted in clinical populations can enhance our understanding of complex neurological disorders (Hallquist & Hillary, 2019), and studies have begun utilizing DCD methods to characterize the spatiotemporal basis of how networks reconfigure across diseases such as epilepsy and autism spectrum disorder (Braun et al., 2016; Martinet et al., 2020). Here, using similar methods, we demonstrate that hormone fluctuations are associated with significant reorganization of the DMN and increased flexibility among several brain networks. Notably, differences in DMN rs-fc emerge among individuals with depression (Greicius et al., 2007) and Alzheimer’s disease (Buckner et al., 2009)—two conditions that display a sex-skewed prevalence towards women (Nebel et al., 2018). Using the MyConnectome Project, Betzel and colleagues provided evidence that increased network flexibility is associated with positive mood (Betzel, Satterthwaite, Gold, & Bassett, 2017). Here, network flexibility was highest during the ovulatory window followed by an immediate decline back to network stability in the luteal phase of the cycle, coincident with traditional rises in negative affect (e.g., premenstrual syndrome; Rubinow & Schmidt, 2006). Although we did not identify a relationship between mood and network flexibility within this participant, cycle-dependent brain network reorganization could play a role in psychiatric conditions observed in some women, particularly those suffering from premenstrual dysphoric disorder. Further, a decline of sex hormones to chronically low states occurs in postreproductive years, decades prior to diagnoses of Alzheimer’s disease. Therefore, modeling time-varying community structure in conjunction with endocrine status could shed light on neurological disorders that display prominent sex differences. If fluctuations in sex hormones lead to greater network flexibility, and those in turn shape the brain and behavior (Betzel et al., 2017), hormone therapy that mimics the transient rise and fall of estradiol could provide a line of treatment for individuals experiencing cognitive symptoms in the transition to menopause and/or for those with a heightened risk for dementia.

### Limitations and Future Directions

The following limitations should be taken into consideration. First, this study involved densely sampling a single female over one complete menstrual cycle, hindering our ability to generalize these findings to other individuals. Therefore, it is critical for this approach to be extended to a larger and more diverse set of women to establish the consistency of these results while accounting for individual differences. Second, we used a well-established group-based atlas to improve generalizability beyond a single-subject design (Schaefer et al., 2018). However, group-based atlases risk loss of individual-level specificity and could overlook meaningful reconfigurations in parcellations (Salehi et al., 2020). Future work using an individual-derived atlas is needed to confirm whether these results are stable across various analytic pipelines. Third, an ongoing debate in network neuroscience surrounds test-retest reliability and what constitutes a “substantial” amount of data per individual. While some studies suggest that (more than 20 min of data per individual is needed (Gratton et al., 2018), others contend that shorter durations (5–15 min) of sampling is sufficient to achieve reliability (Birn et al., 2013; Chen et al., 2015). Repeating this experiment under longer scanning durations (>10 min per day) will be critical for exploring the degree of network stability across the menstrual cycle. Finally, this work considers only between-session rather than within-session network reconfiguration because of the aforementioned concerns about test-retest reliability. However, as previous studies have found meaningful shifts in flexibility across shorter time scales (Braun et al., 2015; Telesford et al., 2016), a natural extension to this work will be to examine within-session network reorganization across the cycle in larger samples of women.

## CONCLUSION

In sum, we demonstrate that resting-state functional connectivity is largely stable within an individual over the course of a complete menstrual cycle. The largest exception to this stability occurs around the ovulatory window, during which peaks in sex hormones result in temporary patterns of brain network reorganization largely localized within areas of the default mode network. Historically, brain-level phenomena resulting from hormone fluctuations have been treated as an unwanted source of variance in population studies and, consequently, studies of this relationship are sparse and underpowered. This work demonstrates that dynamic network methods can reveal important, transient effects of sex hormones that may be overlooked by traditional approaches and provides a novel template for examining the nature of human brain-endocrine relationships.

## METHODS

### 28andMe Experimental Protocol

Data were collected and preprocessed as reported in Pritschet et al. (2020); methods briefly reproduced here. The participant was a right-handed Caucasian female, aged 23 years for the duration of the study. The participant had no history of neuropsychiatric diagnosis, endocrine disorders, or prior head trauma. She had a history of regular menstrual cycles (no missed periods, cycle occurring every 26–28 days) and had not taken hormone-based medication in the 12 months prior to Study 1. The participant gave written informed consent and the study was approved by the University of California, Santa Barbara, Human Subjects Committee.

The participant underwent daily testing for 30 consecutive days, with the first test session determined independently of cycle stage for maximal blindness to hormone status (Study 1). The participant began each test session with a daily questionnaire (9:00 a.m.) followed by a time-locked blood sample collection 10:00 a.m. (±30 min). Endocrine samples were collected, at minimum, after 2 hr of no food or drink consumption (excluding water). This was followed by a 1-hr MRI session (11:00 a.m.) consisting of structural and functional MRI sequences. To note, the participant refrained from consuming caffeinated beverages before each test session. One year later (Study 2), the participant repeated the procedures while on a hormonal regimen (0.02 mg ethinyl-estradiol, 0.1 mg levonorgestrel, Aubra, Afaxys Pharmaceuticals), which she began 10 months prior to the start of data collection (Pritschet et al., 2020).

A licensed phlebotomist inserted a saline-lock intravenous line into the dominant or nondominant hand or forearm daily to evaluate hypothalamic-pituitary-gonadal axis hormones, including serum levels of gonadal hormones (17*β*-estradiol, progesterone, and testosterone) and the pituitary gonadotropins luteinizing hormone (LH) and follicle stimulating hormone (FSH). One 10-ml blood sample was collected in a vacutainer SST (BD Diagnostic Systems) each session. The sample clotted at room temperature for 45 min until centrifugation (2,000 g for 10 min) and was then aliquoted into three 1-ml microtubes. Serum samples were stored at −20°C until assayed. Serum concentrations were determined via liquid chromatography-mass spectrometry (for all steroid hormones) and immunoassay (for all gonadotropins) at the Brigham and Women’s Hospital Research Assay Core.

### fMRI Data Acquisition and Preprocessing

The participant underwent a daily magnetic resonance imaging scan on a Siemens 3T Prisma scanner equipped with a 64-channel phased-array head coil. First, high-resolution anatomical scans were acquired using a T1-weighted magnetization prepared rapid gradient echo (MPRAGE) sequence (TR = 2,500 ms, TE = 2.31 ms, TI = 934 ms, flip angle = 7°; 0.8-mm thickness) followed by a gradient echo fieldmap (TR = 758 ms, TE1 = 4.92 ms, TE2 = 7.38 ms, flip angle = 60°). Next, the participant completed a 10-min resting-state fMRI scan using a T2-weighted multiband echo-planar imaging (EPI) sequence sensitive 468 to the blood oxygenation level–dependent (BOLD) contrast (TR = 720 ms, TE = 37 ms, flip angle = 56°, multiband factor = 8; 72 oblique slices, voxel size = 2 mm). In an effort to minimize motion, the head was secured with a custom, 3D-printed foam head case (https://caseforge.co/) (Days 8–30 of Study 1). Overall motion (mean framewise displacement) was negligible, with fewer than 130 *μ*m of motion on average each day.

Initial preprocessing was performed using the Statistical Parametric Mapping 12 software (SPM12, Wellcome Trust Centre for Neuroimaging, London) in MATLAB. Functional data were realigned and unwarped to correct for head motion and the mean motion-corrected image was coregistered to the high-resolution anatomical image. All scans were then registered to a subject-specific anatomical template created using Advanced Normalization Tools (ANTs) multivariate template construction. A 5-mm full-width at half-maximum (FWHM) isotropic Gaussian kernel was subsequently applied to smooth the functional data. Further preparation for resting-state functional connectivity was implemented using in-house MATLAB scripts. Global signal scaling (median = 1,000) was applied to account for fluctuations in signal intensity across space and time, and voxelwise time series were linearly detrended. Residual BOLD signal from each voxel was extracted after removing the effects of head motion and five physiological noise components (cerebrospinal fluid + white matter signal). Motion was modeled using a Volterra expansion of translational/rotational motion parameters, accounting for autoregressive and nonlinear effects of head motion on the BOLD signal. All nuisance regressors were detrended to match the BOLD time series.

Functional network nodes were defined based on a 400-region cortical parcellation and 15 regions from the Harvard–Oxford subcortical atlas. For each day, a summary time course was extracted per node by taking the first eigenvariate across functional volumes. These regional time series were then decomposed into several frequency bands using a maximal overlap discrete wavelet transform. Low-frequency fluctuations in wavelets 3–6 (0.01–0.17 Hz) were selected for subsequent connectivity analyses. Finally, we estimated the spectral association between regional time series using magnitude-squared coherence: this yielded a 415×415 functional association matrix each day, whose elements indicated the strength of functional connectivity between all pairs of nodes (FDR-thresholded at *q* < 0.05).

### Dynamic Community Detection and Analysis

A multilayer tensor (415 × 415 × 30) was constructed from the association matrices described above for network analysis. Each layer corresponded to the strictly positive, weighted, FDR-thresholded rs-fc association matrix for the corresponding day of the experiment. Interlayer links were added only between adjacent layers. Communities in resting-state connectivity were identified by maximizing multislice modularity, given by

$$Q=\frac{1}{2\mu }\underset{ijlr}{\sum }((A_{ijl}-\gamma_{l}\frac{k_{il}k_{jl}}{2m_{l}})\delta_{lr}+\delta_{ij}\omega_{jlr})\delta (g_{il},g_{jr}),$$ (1)

where *μ* is the total edge weight in the network, *i* and *j* index nodes in slices *l* and *r*, *A* is the adjacency matrix containing edge weights between nodes and slices, *γ* is the structural resolution parameter, *k*_*il*_ is the strength of node *i* in slice *l*, *m*_*l*_ is the total nodal strength in slice *l*, *δ* is the Kronecker delta, *ω* is the temporal resolution parameter, and *g* is the community assignment index (Bassett et al., 2013).

Community assignments that maximize modularity were determined 150 times over a grid of parameter values (*γ*, *ω*) = [0.97, 1.07] × [0.8, 1.5] using the genlouvain function from Jeub et al. in MATLAB 2019a (Jeub, Bazzi, Jutla, & Mucha, 2011–2019). From these community assignments, the consensus partition for each parameter combination was determined using the consensus_similarity function from the Network Connectivity Toolbox (NCT, http://commdetect.weebly.com/).

Node flexibility is defined as the proportion of times a node changes community assignment out of all possible opportunities to change its assignment. Thus, a flexibility value of 1 indicates that a node changes community membership at every time step and a value of 0 indicates that it never changes communities. Partition significance, node flexibility, and persistence were also calculated using functions from the NCT (Bassett et al., 2011). Cross-covariance values were calculated and statistical tests were performed using built-in MATLAB functions.

Head motion was low (< 130 *μ*m), was not significantly associated with hormone concentrations (all pairwise Pearson correlations > 0.05 after Bonferroni correction), and was nearly identical between Days 22 and 24 of the experiment (when reorganization occurred), suggesting that head motion is not a confounding factor when considering community reconfiguration. On the day of the experiment with the fewest connections (Day 26), the network had an edge density of 0.9317 (i.e., 93.17% of possible edges have nonzero values) and the median density was 0.9713. This represents a 4% difference in density, and density was not significantly correlated with hormone levels, so we do not believe the community detection algorithm was biased by disparities in edge density.

## ACKNOWLEDGMENTS

Thanks to Mario Mendoza for phlebotomy and MRI assistance. We would also like to thank Evan Layher, Shuying Yu, Courtney Kenyon, Maggie Hayes, and Morgan Fitzgerald for assistance with data collection.

## SUPPORTING INFORMATION

Supporting information for this article is available at https://doi.org/10.1162/netn_a_00169.

## AUTHOR CONTRIBUTIONS

Joshua M. Mueller: Formal analysis; Investigation; Methodology; Validation; Visualization; Writing - Original Draft; Writing - Review & Editing. Laura Pritschet: Conceptualization; Visualization; Writing - Original Draft; Writing - Review & Editing. Tyler Santander: Data curation; Methodology. Caitlin M. Taylor: Writing - Review & Editing. Scott T. Grafton: Methodology; Supervision; Writing - Review & Editing. Emily Goard Jacobs: Conceptualization; Funding acquisition; Investigation; Supervision; Writing - Review & Editing. Jean M. Carlson: Methodology; Supervision; Writing - Review & Editing.

## FUNDING INFORMATION

Emily G. Jacobs, Brain and Behavior Research Foundation (http://dx.doi.org/10.13039/100000874). Emily Jacobs, California Nanosystems Institute. Emily G. Jacobs, Hellman Family Fund. Emily G. Jacobs, National Institute on Aging (NIH R01 AG063843). Scott T. Grafton, Rutherford B. Fett Fund. Jean M. Carlson, David and Lucile Packard Foundation (http://dx.doi.org/10.13039/100000008). Jean M. Carlson, Institute for Collaborative Biotechnologies (http://dx.doi.org/10.13039/100014543).

## TECHNICAL TERMS

Dynamic community detection: Network science technique that identifies strongly connected sets of nodes (communities) that persist or change over time.
Estradiol: Refers to 17β-estradiol, the major form of estrogen in mammals.
Dense sampling: Experimental paradigm in which phenotypic measures are repeatedly collected within an individual over time.
Flexibility: Measure of how often nodes change communities.
Partition: Set of community assignments for all nodes resulting from dynamic community detection.
