# Supplementary material for: Dynamic community detection reveals transient reorganization of functional brain networks across a female menstrual cycle
Source: Netw Neurosci. 2021 Feb 1;5(1):125–44. doi: 10.1162/netn_a_00169 (PMC7935041; doi:10.1162/netn_a_00169)
Supplement: Supplementary file 1 [file netn-05-125-s001.pdf]

Mueller, J. M., Pritschet, L., Santander, T., Taylor, C. M., Grafton , S. T., Jacobs, E. G. & Carlson, J. M. (2021). Supporting information for "Dynamic community detection reveals transient reorganization of functional brain networks across a female menstrual cycle." *Network Neuroscience*, 5(1), 125–144. https://doi.org/10.1162/netn_a_00169

Supplemental figures

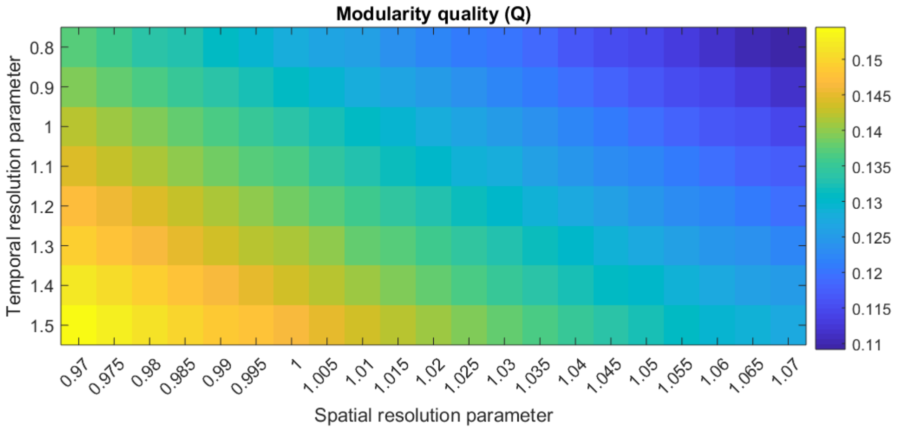

S1 Fig. Resting-state networks are modular over a wide range of community detection parameter choices. Shown is a heat map of the mean modularity quality value (referred to as Q in the main Methods text) of 150 runs of the dynamic community detection algorithm for each parameter combination considered here. Color indicates the value of Q, which ranges from -1 to 1, with a value of 0 indicating a lack of modular structure. While no cutoff exists to identify "true" modular structure per se, higher non-zero positive values of Q indicate more pronounced modularity. Across the parameters considered here, the value of Q ranges from approximately 0.11 to 0.15, indicating weak, but present, modular structure. Consensus partitions are similar across these parameter choices and the networks analyzed here were weighted, undirected, and dense; these factors explain the consistency of Q values.

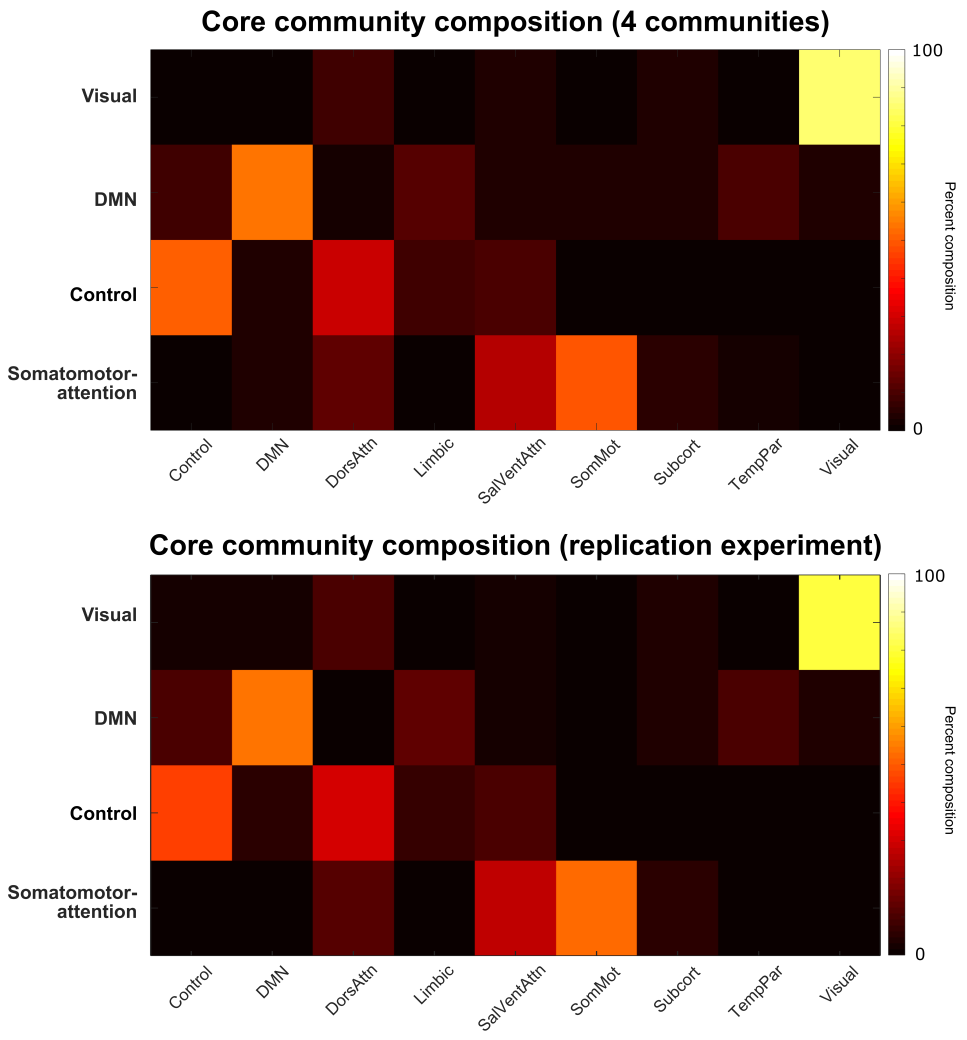

S2 Fig. Core community compositions are consistent across dynamic community detection parameter choices. Shown are heat maps of the core community compositions for the original (top) and follow-up (bottom) experiments. In each case, community compositions are averaged over the set of parameter combinations which result in four communities being identified ($.8 < \omega < 1.5$, $\gamma < 1.015$). The compositions shown here are nearly identical to those shown in Fig. 3A, which is the core community composition identified at the standard parameter combination for the dynamic community detection algorithm ($\omega = \gamma = 1$).

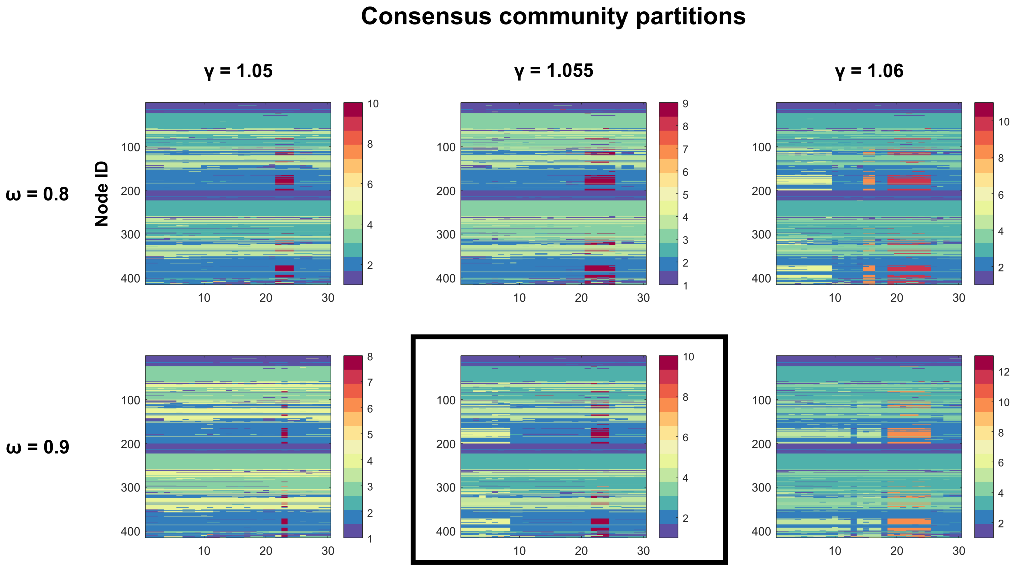

S3 Fig. Functional communities exhibit transient reorganization around the ovulatory window over a range of dynamic community detection parameter values. Shown are the consensus community partitions for a range of dynamic community detection algorithm parameters ($.8 \leq \omega \geq .9$, $1.05 \leq \gamma \geq 1.06$). Y-axis values indicate node identity, x-axis values indicate the day of experiment, and color indicates community membership. The partition outlined in black (bottom middle) is the basis of the quantitative analysis in the main text and is shown in Fig. 4B. Ovulation occurs on day 23 (black underline in bottom middle panel). In each case displayed here, a new subcommunity with a consistent composition splits from the default mode core during the ovulatory window but rejoins after day 25 at the latest, concurrent with a sharp decline in estradiol, LH, and FSH (Fig. 6B). This indicates the presence of a stable, reliable solution to the dynamic community detection algorithm in this parameter range.

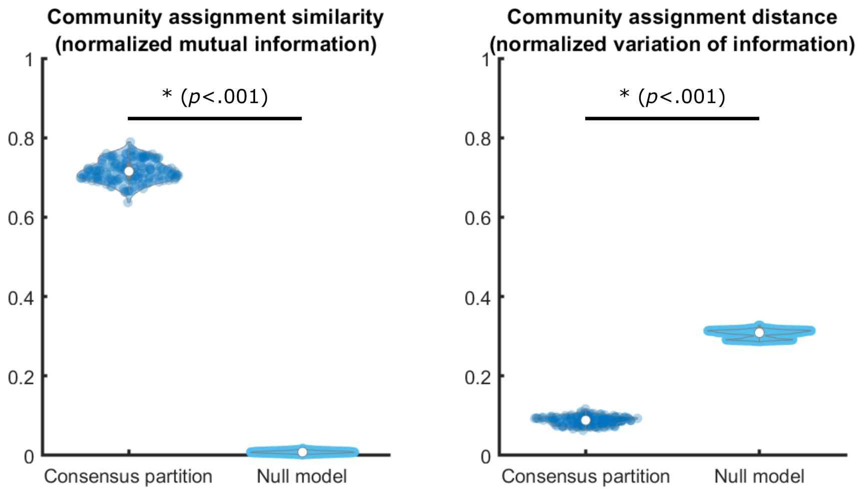

S4 Fig. Solutions to the dynamic community detection algorithm are similar and reliable. Since the Louvain algorithm is non-deterministic, it is important to characterize the stability/similarity of community detection solutions. To compare the consensus partition used for analysis in the main text (illustrated in Figure 4) to results from each run of the dynamic community detection algorithm, we used normalized mutual information (NMI) as a measure of partition similarity and normalized variation of information (NVI) as a measure of partition distance, per Meila et al. (2007). NMI ranges from 0 to 1, with a value of 0 indicating similarity between partitions at no better than chance and 1 indicating a perfect match. NVI ranges from 0 to 1 as well, but with an inverse interpretation (i.e. smaller distances indicate higher similarity). Pairwise NMI and NVI values were calculated between the consensus partition and each output of the 150 runs of the dynamic community detection algorithm. Pairwise NMI and NVI values were also calculated between 100 repetitions of a null model consensus partition, which permuted node identities, and each DCD output. This null model permutation preserves the flexibility trends and community sizes across the experiment, but shuffles node identity to provide a baseline hypothesis against which to test the significance of the true community detection results. The NMI and NVI distributions are significantly higher and lower, respectively, than the null model comparison, indicating that the consensus partition used in subsequent analysis is much better than what would be arrived at by chance. The median NMI between the consensus partition and each DCD algorithm output is 0.72 and the median NVI is 0.09, indicating that the consensus partition is highly representative of each individual output partition from the 150 runs. Therefore, the community detection solution is highly stable and solutions are highly similar.

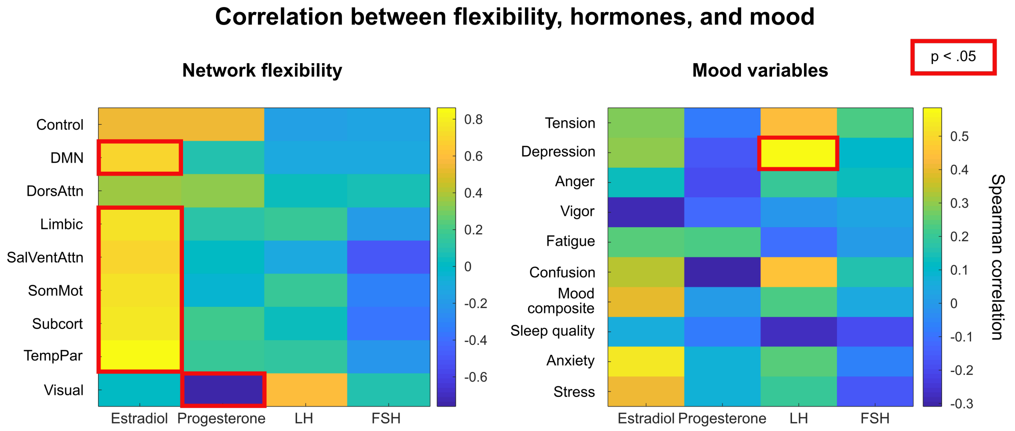

S5 Fig. Network flexibility is significantly correlated with estradiol. Shown on the left is a heat map of the Spearman correlation values between the flexibility and hormone curves shown in Figure 6 of the main text. Color indicates correlation values and red outlines indicate statistical significance at the $p < .05$ level after Bonferroni correction. All networks except the control, dorsal attention, and visual networks have correlation values > 0.6 with estradiol, indicating that these variables are tightly coupled. The visual network also had a statistically significant negative correlation with progesterone, but this network was minimally flexible so the effect size was negligible. No other hormone-flexibility relationships were significant, suggesting that estradiol is the primary driver of flexibility. On the right, a heat map illustrates the minimal significant relationships between sex hormones and mood variables, which were surveyed each day of the experiment (see Pritschet et al., 2020). Flexibility and mood values were not significantly correlated for any networks or mood variables.

Node Label	Network	Subnetwork	xMNI	yMNI	zMNI
17Networks_LH_DefaultB_Temp_2	DMN	DMN B	-54	-2	-30
17Networks_LH_DefaultB_Temp_3	DMN	DMN B	-62	-18	-20
17Networks_LH_DefaultB_Temp_4	DMN	DMN B	-56	-8	-14
17Networks_LH_DefaultB_Temp_5	DMN	DMN B	-60	-34	-4
17Networks_LH_DefaultB_Temp_6	DMN	DMN B	-52	-22	-6
17Networks_LH_DefaultB_IPL_1	DMN	DMN B	-46	-58	20
17Networks_LH_DefaultB_IPL_2	DMN	DMN B	-56	-54	30
17Networks_LH_DefaultB_PFCd_1	DMN	DMN B	-4	52	28
17Networks_LH_DefaultB_PFCd_2	DMN	DMN B	-14	58	30
17Networks_LH_DefaultB_PFCd_3	DMN	DMN B	-22	50	32
17Networks_LH_DefaultB_PFCd_4	DMN	DMN B	-8	42	52
17Networks_LH_DefaultB_PFCd_5	DMN	DMN B	-12	24	60
17Networks_LH_DefaultB_PFCd_6	DMN	DMN B	-6	10	64
17Networks_LH_DefaultB_PFCl_1	DMN	DMN B	-40	20	48
17Networks_LH_DefaultB_PFCl_2	DMN	DMN B	-42	8	48
17Networks_LH_DefaultB_PFCv_1	DMN	DMN B	-36	22	-16
17Networks_LH_DefaultB_PFCv_2	DMN	DMN B	-36	36	-12
17Networks_LH_DefaultB_PFCv_3	DMN	DMN B	-46	32	-10
17Networks_LH_DefaultB_PFCv_4	DMN	DMN B	-48	28	0
17Networks_LH_DefaultB_PFCv_5	DMN	DMN B	-54	20	12
17Networks_RH_DefaultB_Temp_1	DMN	DMN B	64	-24	-8
17Networks_RH_DefaultB_Temp_2	DMN	DMN B	64	-38	0
17Networks_RH_DefaultB_AntTemp_1	DMN	DMN B	50	8	-32
17Networks_RH_DefaultB_PFCd_1	DMN	DMN B	6	58	30
17Networks_RH_DefaultB_PFCd_2	DMN	DMN B	16	52	36
17Networks_RH_DefaultB_PFCd_3	DMN	DMN B	4	44	40
17Networks_RH_DefaultB_PFCd_4	DMN	DMN B	14	38	52
17Networks_RH_DefaultB_PFCd_5	DMN	DMN B	12	20	62
17Networks_RH_DefaultB_PFCv_1	DMN	DMN B	34	22	-18
17Networks_RH_DefaultB_PFCv_2	DMN	DMN B	48	32	-8
17Networks_RH_DefaultB_PFCv_3	DMN	DMN B	54	24	6
17Networks_LH_ContC_pCun_1	Control	Control C	-10	-70	32
17Networks_LH_ContC_pCun_2	Control	Control C	-10	-78	46
17Networks_LH_ContC_pCun_3	Control	Control C	-4	-64	52
17Networks_LH_ContC_Cingp_1	Control	Control C	-6	-40	24
17Networks_LH_ContC_Cingp_2	Control	Control C	-4	-22	30
17Networks_RH_ContC_pCun_1	Control	Control C	16	-64	28
17Networks_RH_ContC_pCun_2	Control	Control C	14	-72	40
17Networks_RH_ContC_pCun_3	Control	Control C	6	-64	44
17Networks_RH_ContC_pCun_4	Control	Control C	8	-50	44
17Networks_RH_ContC_pCun_5	Control	Control C	8	-72	52
17Networks_RH_ContC_Cingp_1	Control	Control C	8	-44	20
17Networks_RH_ContC_Cingp_2	Control	Control C	6	-28	28

S6 Fig. Node identities from the Schaefer functional-anatomical atlas. Shown here are the node identities for regions in the Control C and DMN B subnetworks. Control C nodes are identified as being highly flexible over the entire course of the menstrual cycle. Within-network connectivity between nodes in DMN B increases around the ovulatory window, resulting in a transient bifurcation of the default mode core community (Fig. 5).

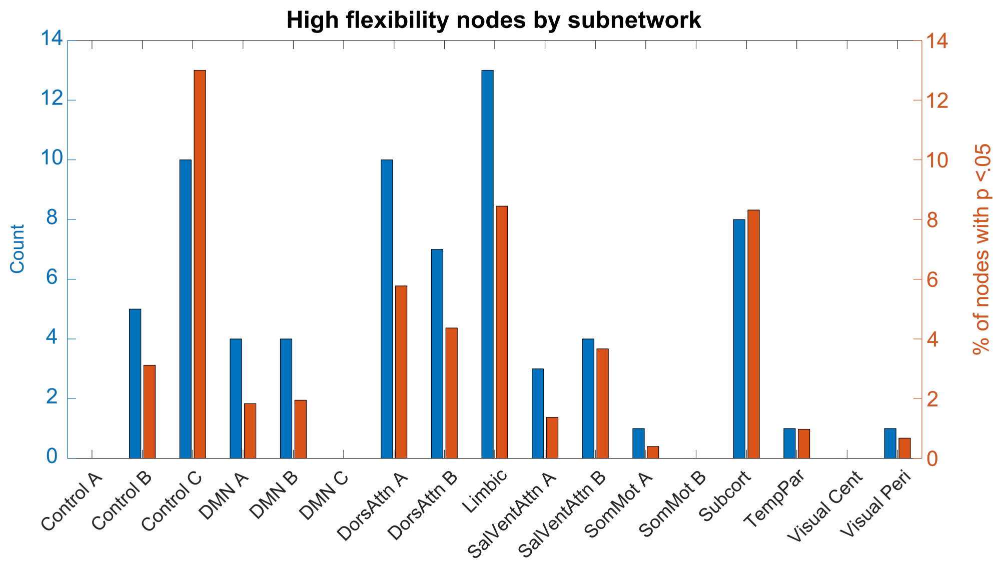

S7 Fig. Functional-anatomic subnetworks have distinct flexibility profiles. Within functional-anatomical networks, nodes belonging to different subnetworks exhibit different flexibility trends. Specifically, Control C subnetwork nodes are the most likely to be highly flexible within the Control network, suggesting a specific "integrator" role for this subnetwork.

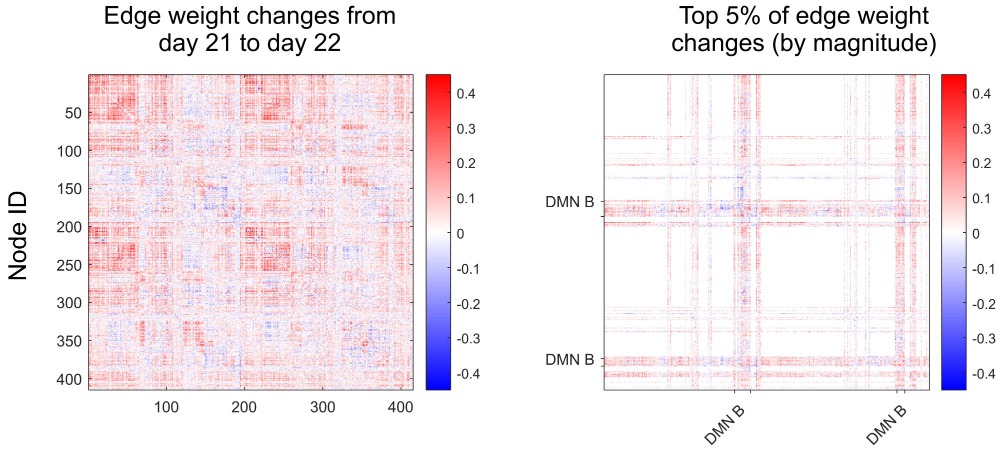

S8 Fig. Strong changes in functional connectivity during the ovulatory window are localized to a default mode subnetwork. Shown here are the differences in edge weights (magnitude-squared coherence values) between nodes on days 21 and 22, when reorganization took place, with color indicating value. Positive values indicate an increase in edge weight from day 21 to 22. On the right, only the top 5% of changes by magnitude are displayed.

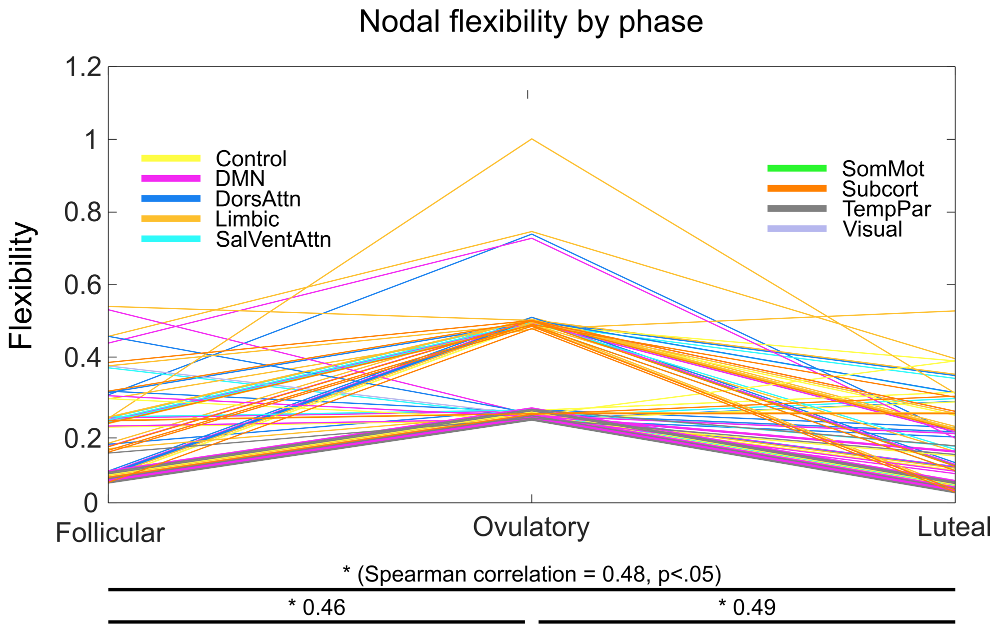

S9 Fig. Highly flexible nodes are consistently flexible across the menstrual cycle, but exhibit peak flexibility during the ovulatory window. Shown here are the flexibility values of the 104 nodes with non-zero flexibility in each of the three menstrual cycle phases. Color indicates functional-anatomical network membership. Spearman rank correlation coefficients between phases are shown below the plot; all three phases exhibit moderate correlation with one another, indicating that high and low flexibility nodes are consistently highly or less flexible, respectively, across the menstrual cycle.

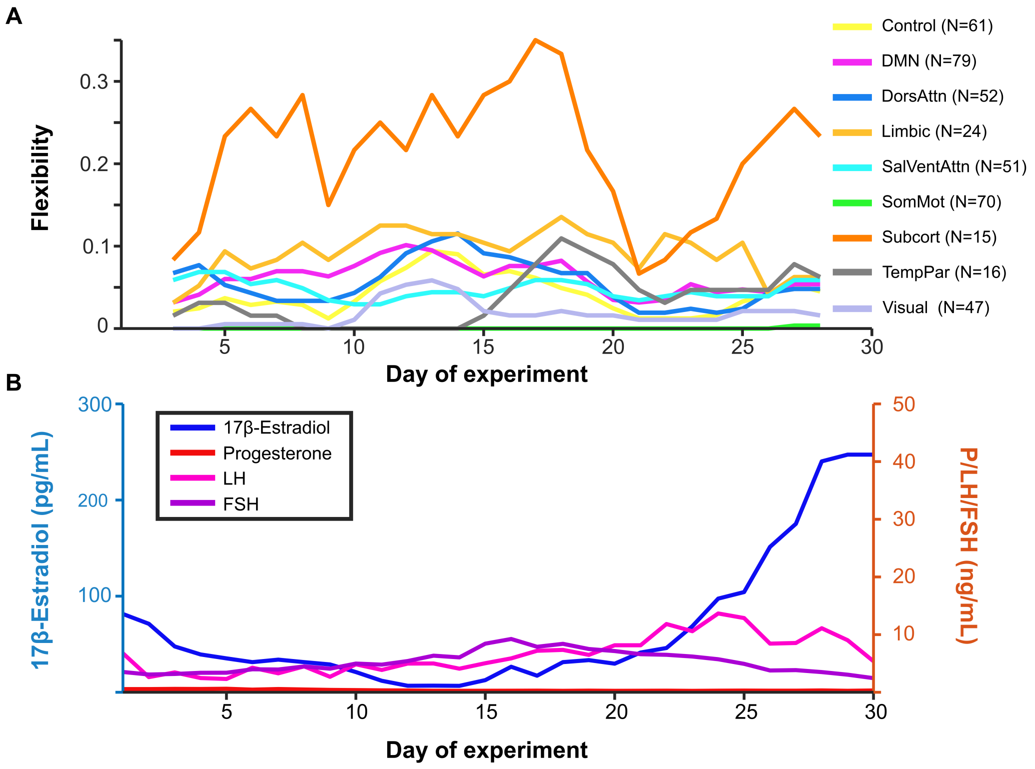

S10 Fig. Network flexibility is reduced in a follow-up oral hormonal contraceptive replication experiment. When endogenous hormone levels were modulated via oral contraceptive administration in a replication experiment, flexibility levels did not increase concurrent with the rise in estradiol normally associated with ovulation, as observed in the naturally-cycling condition (Figure 6). Shown here are curves for the mean flexibility of each network calculated over a 5-day sliding window for the parameter combination $(\omega,\gamma) = (0.9,1.055)$ (the same parameters used for dynamic community detection in the main text). It should be noted that the experiment concluded before estradiol levels subsided to baseline concentrations, so further experiments are needed to validate this result.

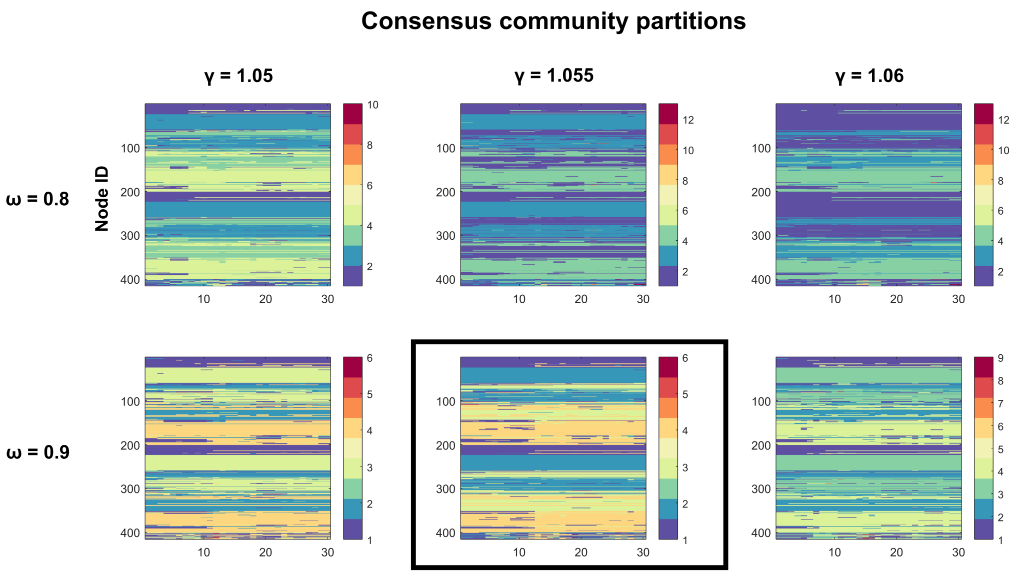

S11 Fig. Brain networks do not exhibit reorganization in an oral hormonal contraceptive replication experiment. Shown here are the consensus partitions for a replication experiment in which the same participant underwent a hormonal regimen via oral contraceptive administration. Under this regimen, no large-scale reorganization events (like those observed in Figure 3) occurred, strengthening the conclusion that coordinated sex hormone fluctuations experienced across the menstrual cycle are responsible for the changes in connectivity observed in the main experiment.

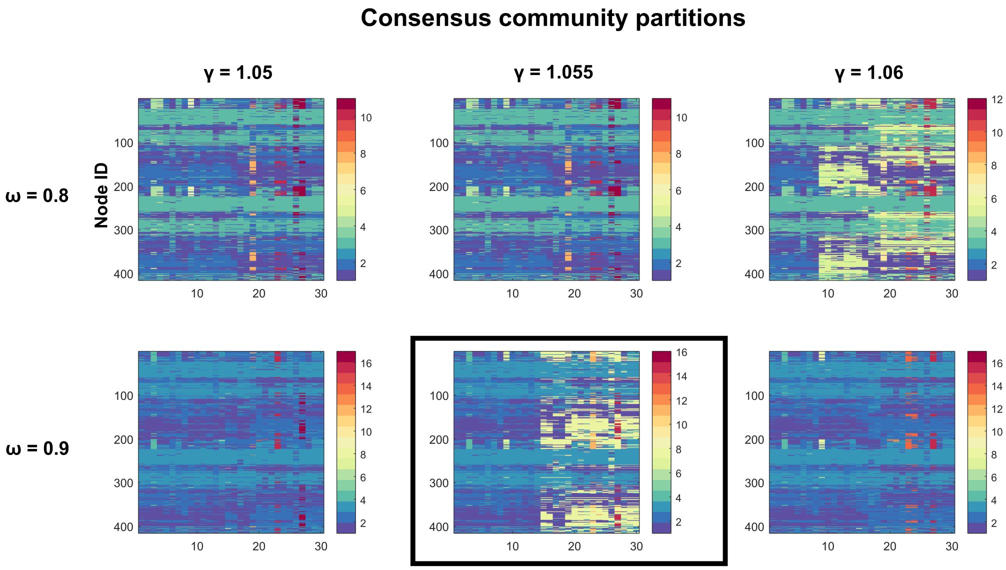

S12 Fig. Dynamic community detection is less robust to correlation networks than coherence networks. Shown here are the consensus partitions for the weighted, signed FC networks using Pearson correlation as edge weights. These partitions were more fragmented and less stable than those observed when using coherence as the FC measure (compare to Supplementary Figure 3).

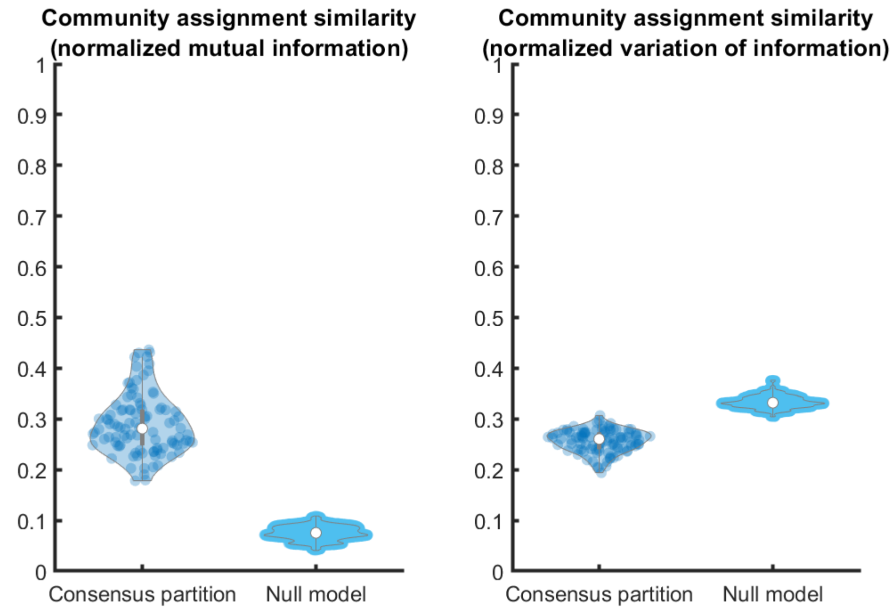

S13 Fig. Dynamic community detection solutions are less similar in correlation networks than coherence networks. Solutions to the dynamic community detection algorithm are less consistent when using Pearson correlation as the FC measure, indicating poor community detection algorithm performance (compare to Supplementary Figure 4).
